# Troubleshooting techniques for the Endurant^TM^ device in endovascular aortic aneurysm repair

**DOI:** 10.5830/CVJA-2014-049

**Published:** 2014

**Authors:** George S Georgiadis, Efstratios I Georgakarakos, Christos Argyriou, Miltos K Lazarides, George A Antoniou, George Trellopoulos

**Affiliations:** Department of Vascular Surgery, Demokritus University of Thace, University General Hospital of Alexandroupolis, Greece; Department of Vascular Surgery, Demokritus University of Thace, University General Hospital of Alexandroupolis, Greece; Department of Vascular Surgery, Demokritus University of Thace, University General Hospital of Alexandroupolis, Greece; Department of Vascular Surgery, Demokritus University of Thace, University General Hospital of Alexandroupolis, Greece; Department of Vascular Surgery, Red Cross Hospital of Athens, Greece; Surgical Sector, Vascular Surgery Unit, Georgios Papanikolaou Hospital, Thessaloniki, Greece

**Keywords:** abdominal aortic aneurysm, endovascular aortic aneurysm repair, stent-graft, Endurant device, techniques

## Abstract

Endovascular aortic aneurysm repair with the Endurant^TM^ stent-graft system has been shown to be safe and effective in high-risk surgical patients with complex suprarenal and/or infrarenal abdominal aortic aneurysm anatomy. The wireformed M-shaped stent architecture and proximal springs with anchoring pins theoretically permit optimal sealing in shorter and more angulated proximal aneurysm necks even under off-label conditions. Nonetheless, extremely difficult anatomical situations and inherent graft system-related limitations must be anticipated. Herein, we describe our techniques to overcome the capture of the tip sleeve within the suprarenal bare-stent anchoring pins, other endograft segments, and native vessels.

## Abstract

Previous randomised trials have confirmed the short and mid-term benefits of endovascular abdominal aortic aneurysm repair versus open repair.^1^,^2^ However, its success is dependent on specific anatomical parameters that include the abdominal aortic aneurysm (AAA) morphology and dimensions. Adverse anatomical characteristics such as very short and severely angulated proximal aortic necks or small and tortuous iliac arteries can occasionally preclude its use. Advances in AAA endograft device technology have significantly contributed to improved patient outcomes, and durability of the procedure allows for a wider therapeutic spectrum of patients to receive endovascular repair (EVAR).

The success of these new stent-graft devices results from better adaptation and improved performances in challenging anatomies and better trackability of delivery systems.^3^-^8^ Specific advancements include improved tip design and greater flexibility, controlled proximal stent-graft release mechanism with re-positional proximal stent-graft capabilities, and improved deliverability and placement accuracy.^3^-^9^ These technological advances, combined with cumulative physician clinical experience and enhanced skill sets, have resulted in the consideration of endoluminal grafting in off-label conditions.

A recent report highlighted the application of troubleshooting techniques to overcome ‘pitfalls’ in some of the steps of EVAR with the Endurant^TM^ (Medtronic Cardiovascular, Santa Rosa, CA) stent-graft device.^10^ Herein, we specifically describe simple techniques to overcome capture of the Endurant^TM^ tip sleeve within the suprarenal bare-stent anchoring pins or within other endograft and native vessel segments, in order to avoid emergency conversion to open repair and the potential for adverse outcomes.

## The Endurant^TM^ stent-graft system

This stent-graft is a new fourth-generation device comprising a high-density multifilament polyester graft material of low porosity, externally supported by an electropolished nitinol stent structure and loaded in a low-profile hydrophilic coating delivery system. The seals of the European Union (EU) as well as Food and Drug Administration (FDA) approval for this device were received in July 2008 and December 2010, respectively.

The Endurant^TM^ stent-graft is designed to enhance performance in AAA patients with straightforward (friendly) or challenging (hostile) anatomies. Its high flexibility and conformability enables the device to adapt to straight as well as severely tortuous proximal aortic necks and challenging iliac artery anatomies. These stent-grafts have a sinusoidal M-shaped architecture with a small amplitude providing optimal sealing in short and angulated proximal aneurysm necks. Furthermore, the M-shaped proximal stent at the upper pole of the endograft body facilitates enhanced wall apposition, minimising the risk of in-folding and providing another 5 mm of sealing zone.

The Endurant^TM^ stent-graft relies on proximal active fixation, incorporating a suprarenal bare stent ring with anchoring pins of increased flexibility, compared to earlier generation stentgrafts. Initially covered by the tip sleeve, the suprarenal stent with anchoring pins provides controlled release and secure fixation. The radiopaque markers at the proximal and distal edges of the stentgraft as well as the flow divider and contralateral gate markers ensure accurate positioning of the device. Apart from a more flexible main body, the limb stent and optimal stent spacing offer more distal longitudinal flexibility and are designed to prevent kinking and provide refined adaptation to tortuous iliac arteries.

Finally, the graft delivery system is reduced by approximately 3 French (Fr) sizes from the smallest prior endograft delivery system. It is available in outer diameters from 18- to 20-Fr for the main body and from 14- to 16-Fr for the extensions. Bifurcated main body proximal diameters include sizes of 23, 25, 28, 32 and 36 mm; limb diameters include sizes of 10, 13, 16, 20, 24 and 28 mm. The diameter of the stent-graft is oversized by approximately 20% in relation to the outer aortic diameter at the proximal fixation zone and about 10% in the distal landing zones (usually the common iliac arteries).

Recently, renovation of the Endurant^TM^ system has resulted in an improved version. Endurant® II provides three additional advanced design features: (1) a 35% extended hydrophilic coating allows the 28-mm-diameter bifurcated component to fit inside an 18-Fr outer diameter catheter (initially 20-Fr with the original Endurant); (2) availability of two new contralateral limb lengths (156 and 199 mm) enables more configuration options and requires fewer total components; and (3) improved radiopacity of the distal end of the bifurcated component’s contralateral gate increases visibility. The Endurant® II device received FDA approval in June 2012.^11^ The following technical scenarios are also applicable to Endurant® II.

## Technical notes

## Scenario 1: Capture of the tip sleeve within the suprarenal bare-stent anchoring pins

This scenario assumes that the main body of the bifurcated component of the Endurant^TM^ stent-graft is deployed and the delivery system advanced proximally as far as 3 cm apart from the suprarenal stent [see manufacture instructions for use (IFU) for system details]. The next step is very crucial and failure to withdraw the delivery system until the spindle is retracted into the fabric portion of the stent-graft results in trapping of a suprarenal crown within the tapered tip sleeve.

**Fig. 1.**
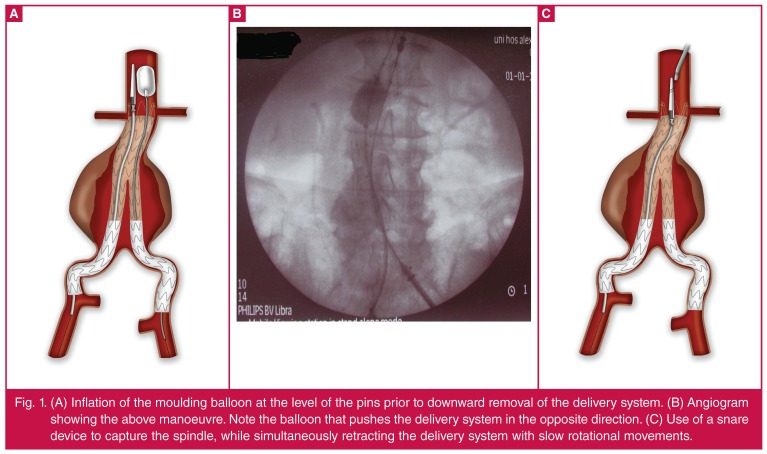
(A) Inflation of the moulding balloon at the level of the pins prior to downward removal of the delivery system. (B) Angiogram showing the above manoeuvre. Note the balloon that pushes the delivery system in the opposite direction. (C) Use of a snare device to capture the spindle, while simultaneously retracting the delivery system with slow rotational movements.

Even though the steps described in the IFU for the Endurant^TM^ stent-graft system may be followed accurtely, in some cases, especially severe angulated necks (≥ 60°), the markedly flexible delivery system will follow the aortic configuration and stack within the hooks of the suprarenal stent. To avoid the need for open conversion, three simple techniques to successfully remove the delivery system of this endograft are described:

• The first action is to completely remove the stiff or superstiff guide wire (usually Amplatz^TM^, Ontract, Archer^TM^ or Lunderquist) inside the delivery system, and then rotationally withdraw the delivery system. Removing the wire allows the graft to follow the natural aortic anatomy. Under straightforward circumstances, the device may bend along the body–ipsilateral endograft, possibly avoiding stacking at the level of the anchoring pins.
• The above manoeuvre might be performed more safely if catheterisation of the docking limb and insertion and deployment of the contralateral limb precedes delivery system withdrawal. Otherwise its removal may be facilitated by keeping the contralateral limb in place while moderately inflating (less than the suprarenal aortic diameter) the molding balloon (e.g. Reliant®, Equalizer or Coda) at the pins’ level prior to downward removal of the delivery system (Fig. 1A, 1B).
• When compelling anatomical conditions exist, another option is to place a large introducer sheath (e.g. Cook 16- or 24-Fr), through the already catheterised docking limb, advancing above the suprarenal stent before delivery system withdrawal. This manoeuvre leads to aorto-iliac axis ‘technical remodelling’ with further proximal neck straightening, a condition that may help to alter the path that the system follows when rotated downwards for removal. The proximal neck might also be straightened after placing a super-stiff or extra-stiff guide wire from the left brachial artery and through the endograft to exit from the contralateral femoral side.
• The last option replaces the guide wire with a snare device that is introduced through a 7-, 12- or 14-Fr sheath via the left brachial artery access, and captures the spindle while simultaneously retracting the delivery system with slow rotational movements (Fig. 1C).

## Scenario 2: The delivery system blocks at the flow divider level

**Fig. 2.**
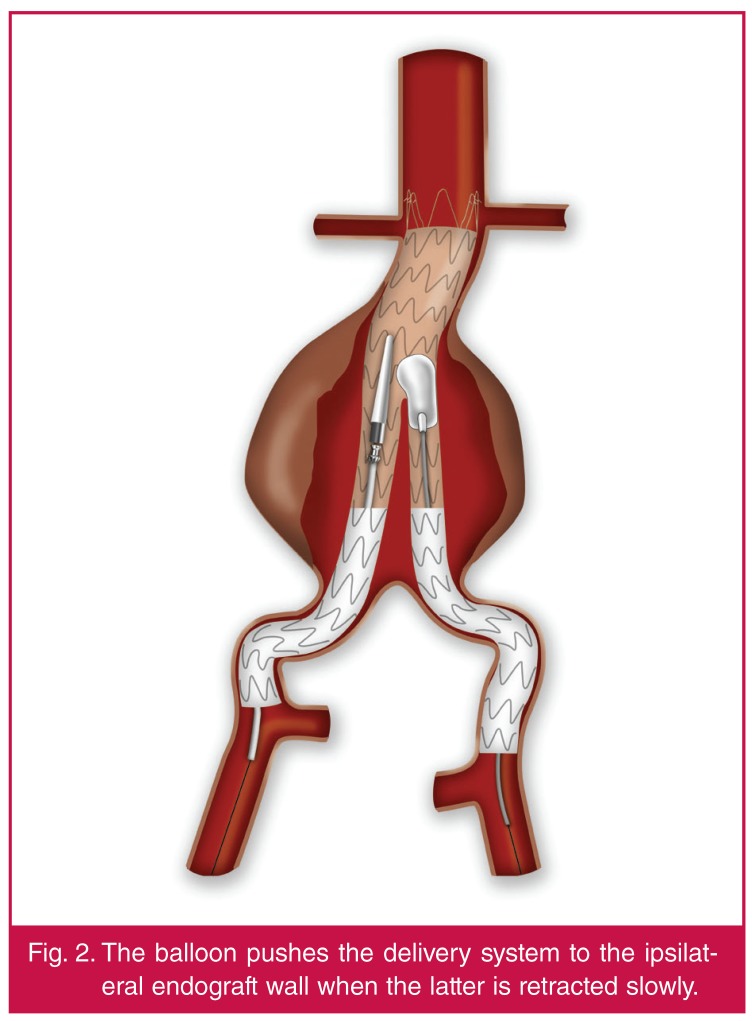
The balloon pushes the delivery system to the ipsilateral endograft wall when the latter is retracted slowly.

In this situation the delivery system moved slightly upwards. The troubleshooting technique includes first deployment of the contralateral limb in the standard fashion, followed by insertion of a moulding balloon (e.g. Reliant®, Equalizer or Coda), which is inflated in the same manner as required to push the delivery system to the ipsilateral endograft wall, when the latter is retracted slowly (Fig. 2).

## Scenario 3: The delivery system blocks at the ipsilateral limb

**Fig. 3.**
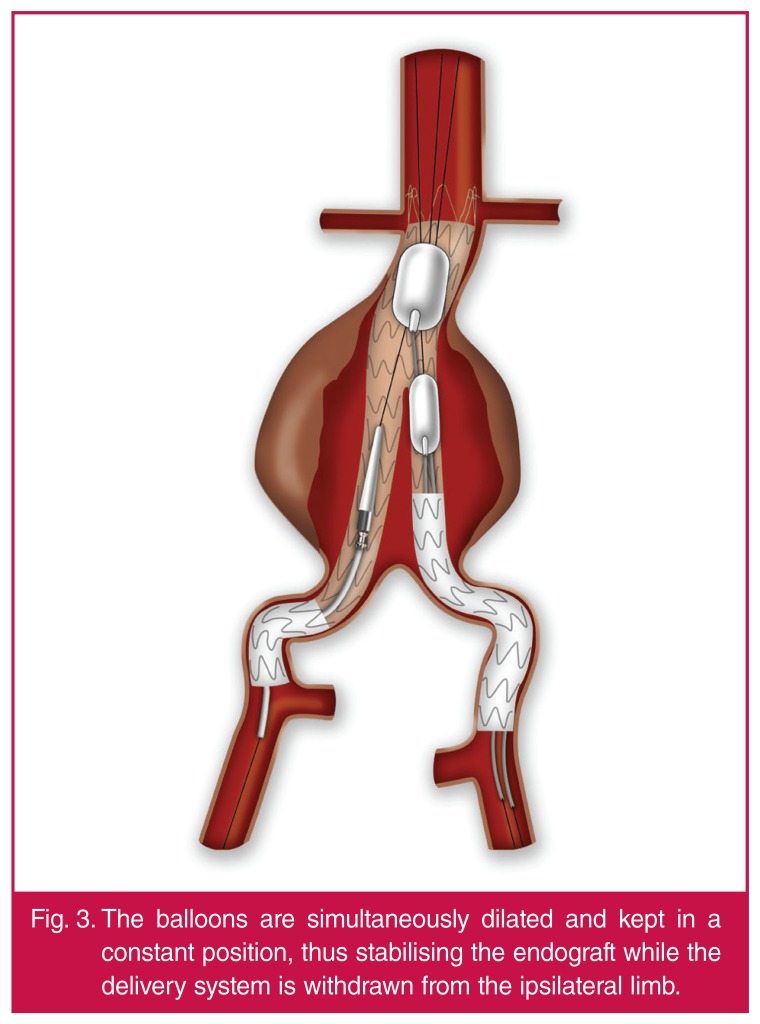
The balloons are simultaneously dilated and kept in a constant position, thus stabilising the endograft while the delivery system is withdrawn from the ipsilateral limb.

Two moulding balloons (e.g. Reliant®, Equalizer or Coda) are required in this situation. They are inserted through a 14-Fr Cook introducer sheath from the contralateral site after the contralateral limb is completely liberated and dilated. One moulding balloon is positioned above the flow divider and the second one at the body-to-contalateral limb overlapping area. They are simultaneously dilated and kept in a constant position, thus stabilising the endograft while the delivery system is withdrawn from the ipsilateral limb (Fig. 3).

The same concept may be applied with a larger balloon coming from above through the left brachial artery. In this case the balloons are inflated and retracted in opposite directions. This bidirectional balloon retraction allows more powerful downward movement of the delivery system.

## Scenario 4: The delivery system blocks at the external iliac artery

**Fig. 4.**
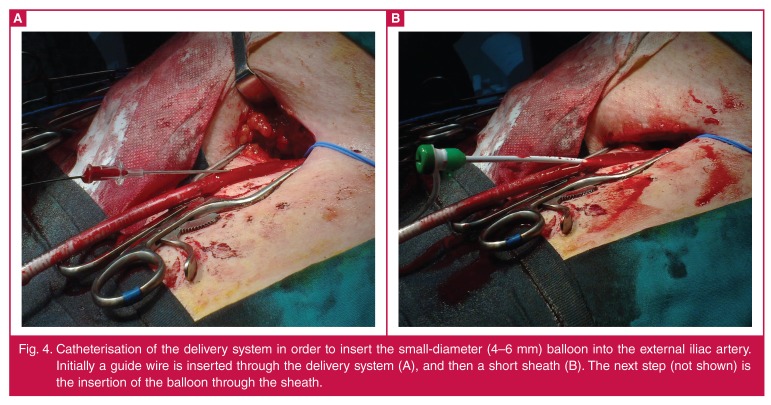
Catheterisation of the delivery system in order to insert the small-diameter (4–6 mm) balloon into the external iliac artery. Initially a guide wire is inserted through the delivery system (A), and then a short sheath (B). The next step (not shown) is the insertion of the balloon through the sheath.

The only way to avoid open conversion in this scenario is to perform a balloon angioplasty of the external iliac artery. Catheterise the delivery system, insert a second 180-cm (0.035-inch) hydrophilic wire between the delivery system and the arterial wall, and place it into the aneurysm sac (Fig. 4A, 4B). A small-diameter (4–6 mm) balloon is introduced over the wire and then into the external iliac artery. Under low pressure, angioplasty is performed. It is not required to fully dilate the balloon up to 8 Atm since the purpose is just to freely remove the delivery system from the stenotic area. In this scenario not only a guide wire, but even a sheath and later a balloon, can be inserted through the delivery system.

## Discussion

Improvements in the endovascular stent-graft design, device delivery and deployment characteristics have all resulted in increased use of EVAR for not only straightforward cases but for those with more complex and challenging aneurysm anatomies. The tips and tricks presented in this report regarding Endurant^TM^ trapped delivery systems should prove especially useful for procedures involving adverse proximal aortic necks and iliac anatomies. It is important to remember that hostile infrarenal aortic aneurysm anatomy such as a very short, severely angulated or dilated proximal neck still remains a major cause of early failure of EVAR and jeopardises long-term efficacy.

Introduction of new endograft devices into the vascular realm will most likely expand the indications for procedures once considered not feasible in the past. Anatomical morphology and measurements of the aneurysm will be crucial to device selection, and device choice critical to the successful positioning and adaptation of the stent-graft to the aneurysm environment for its exclusion from the circulation.

The Endurant^TM^ stent-graft is part of a next-generation system that was designed with the clear intention of expanding the applicability of EVAR for AAA. Initial clinical experience has demonstrated that it can be used in challenging anatomies and can be delivered and deployed safely, even in highly angulated (> 60°) and short (< 15 mm) proximal necks.^5^,^6^ Moreover, accruing experience suggests its safety, even in compelling off-label indications.^7^,^10^,^12^ Despite the fact that durable efficacy of EVAR using the Endurant^TM^ device remains to be demonstrated, intra-operative performance of this endograft in hostile aneurysm morphology adds valuable information to other recently reported clinical short- and mid-term results.^5^-^8^,^10^,^12^

Technical manoeuvres may occasionally be required in difficult anatomies in order to avoid severe complications. Although not confirmed in all Endurant clinical studies,^12^ one problem reported in short and tightly angulated necks is the difficulty of retrieving the conical proximal shelter for the non-covered proximal stent.

In a recent study, comparing the performance of the newly released Edurant II® endograft in patients with friendly and hostile infrarenal aortic anatomy eligible for EVAR, the necessity of troubleshooting techniques was significantly higher in the hostile group.^10^ Herein, we described some of these techniques, including those most frequently encountered, the capture of the tip sleeve within the suprarenal bare-stent anchoring pins.^10^

Its easy, accurate and controlled deployment, coupled with its unique high flexibility and conformability contributes to its successful use, even in severely angulated proximal necks and/or iliac arteries. Friendly and hostile groups had equal performance regarding all primary outcome measures, suggesting that expanded EVAR indications can be applied with this stent-graft.^10^ Knowledge of these described troubleshooting techniques should allow physicians to handle even the most extreme scenarios with the EndurantTM endograft system and other endoprostheses featuring a suprarenal stent with anchors or pins.

## Conclusion

The tips and tricks presented in this report should prevent or reduce conversion to an open procedure when the Endurant^TM^ delivery system becomes trapped in the suprarenal stent anchoring pins or other graft segments. While this report is written specifically for the Endurant^TM^ device system, lessons gleaned are applicable to similar endograft systems.
